# Chronic kidney disease is more prevalent among women but more men than women are under nephrological care: Analysis from six outpatient clinics in Austria 2019

**DOI:** 10.1007/s00508-022-02074-3

**Published:** 2022-08-31

**Authors:** Michal J. Lewandowski, Simon Krenn, Amelie Kurnikowski, Philipp Bretschneider, Martina Sattler, Elisabeth Schwaiger, Marlies Antlanger, Philipp Gauckler, Markus Pirklbauer, Maria Brunner, Sabine Horn, Emanuel Zitt, Bernhard Kirsch, Martin Windpessl, Manfred Wallner, Ida Aringer, Martin Wiesholzer, Manfred Hecking, Sebastian Hödlmoser

**Affiliations:** 1 Department of Internal Medicine III, Clinical Division of Nephrology and Dialysis, Medical University of Vienna, Vienna, Austria; 2 Department of Internal Medicine II, Keplerklinikum Linz, Linz, Austria; 3 Department of Internal Medicine IV (Nephrology and Hypertension), Medical University of Innsbruck, Anichstraße 35, 6020 Innsbruck, Austria; 4 Department of Internal Medicine, Landeskrankhaus Villach, Villach, Austria; 5 Department of Internal Medicine III, Landeskrankenhaus Feldkirch, Feldkirch, Austria; 6 Department of Internal Medicine III, Landesklinikum Mistelbach, Mistelbach, Austria; 7 Department of Internal Medicine IV, Klinikum Wels-Grieskirchen, Wels, Austria; 8 Department of Internal Medicine I, Universitätsklinikum St. Pölten, St. Pölten, Austria; 9 Department of Epidemiology, Center for Public Health, Medical University of Vienna, Vienna, Austria

**Keywords:** CKD, Sex disparity, Outpatient, Outpatient services utilization, CKD-EPI 2021 equation

## Abstract

**Background:**

A discrepancy between sex-specific treatment of kidney failure by dialysis (higher in men) and the prevalence of chronic kidney disease in the general population (higher in women) has been reported internationally, but the prevalence by sex has not been described for Austria. Sex disparity among nephrology outpatients has not been studied.

**Methods:**

We employed two formulae (2009 CKD-EPI suppressing the race factor, and race-free 2021 CKD-EPI) to estimate the sex distribution of CKD in Austrian primary care, based on creatinine measurements recorded in a medical sample of 39,800 patients from general practitioners’ offices (1989–2008). Further, we collected information from all clinic appointments scheduled at nephrology departments of 6 Austrian hospitals (Wien, Linz, Wels, St. Pölten, Villach, Innsbruck) during 2019 and calculated visit frequencies by sex.

**Results:**

Using the 2009 CKD-EPI formula, the prevalence of CKD in stages G3–G5 (estimated glomerular filtration rate < 60 mL/min/1.73 m^2^) was 16.4% among women and 8.5% among men aged > 18 years who had attended general practitioners’ offices in Austria between 1989 and 2008 and had at least one creatinine measurement performed. Using the 2021 CKD-EPI formula, the respective CKD prevalence was 12.3% among women and 6.1% among men. In 2019, 45% of all outpatients at 6 participating nephrology departments were women. The median of nephrology clinic visits in 2019 was two (per year) for both sexes.

**Conclusion:**

CKD is more prevalent among Austrian women than men. Men are more prevalent in nephrology outpatient services. Research into causes of this sex disparity is urgently needed.

## Introduction

In most countries of the world, chronic kidney disease (CKD) is more prevalent among women than men [1]. Specifically, when we summarized data from population-based studies of 21 countries [2–24] for our earlier review article [1], we concluded that despite wide geographical variation, higher CKD prevalence in women compared to men was observed in China, Germany, Tibet, Finland, Korea, Turkey, Singapore, Canada, India, Portugal, Australia, Sweden, Poland, Italy, Spain, the USA, the United Kingdom and France. Only in datasets from Thailand and Japan we found that CKD prevalence was lower in women than men.

However, also consistently around the globe, men comprise most patients receiving kidney replacement treatment in the form of dialysis and kidney transplantation, where we typically observe a 60:40 ratio of men versus women [1, 25–27]. This discrepancy has raised concerns that women with CKD may be at a disadvantage in accessing kidney replacement treatment [28, 29].

Kidney function assessment is most commonly derived from the estimated glomerular filtration rate (eGFR). The two most popular eGFR formulas, the 2006 Modification of Diet in Renal Disease (MDRD) and 2009 CKD-Epidemiology Collaboration (EPI) [30] formulas, are both based on creatinine and include, besides adjustments for age and sex, a correction factor for race (black vs. non-black). Following an ongoing discussion about the adequacy of race adjustments in clinical assessment, the authors of the 2009 CKD-EPI formula recently presented a race-free alternative for GFR estimation. In evaluation datasets, this new equation (which we will refer to as the 2021 CKD-EPI formula), overestimated GFR in non-blacks to a higher extent (median difference of estimated and measured GFR: 3.9 ml/min/1.73 m^2^) than the previous version (median 0.5 ml/min/1.73 m^2^) [31]. It is unclear how suitable the new equation is for the Austrian population, where over 94% of residents are either born in the EU or originate from former Yugoslavia or Turkey [32].

In Austria, nationwide CKD prevalence in 2017 was estimated to be 9.77% [33], using the Global Burden of Disease (GBD) criteria. Current prevalence estimates by sex are not available.

In general, women are believed to seek healthcare more often than men. For instance, women in the USA utilize outpatient [34] and preventive care services [35] more often, and women over 60 years of age in Spain utilize healthcare services more than men in the same age group [36]. Higher prevalence of chronic illness and reproductive health consultations (e.g., pregnancy, childbirth, contraceptives) were identified as causes driving the difference between genders in healthcare utilization [37]. For kidney patients, the literature is more limited. In the USA, women with lupus nephritis are known to utilize outpatient services more than men suffering from the same disease with no difference in outcomes [38].

In our research, we are currently asking whether women with CKD may have different health-seeking behavior than men with the same condition. To gain new insights, we recently conducted a qualitative interview study within Austria (publication pending). The questions asked in this study are centered around men’s and women’s attitudes when confronted with CKD, and also around doctor-patient relationships. As a part of this project, the present analysis sought to fill the knowledge gap that exists for Austria, depicting the sex distribution among 2019 nephrology outpatients and their appointment frequency. Further, we offer CKD prevalence estimates by sex in Austrian primary care, both with the established CKI-EPI 2009 formula and its race-free 2021 alternative. The results can provide further context for research on sex and gender differences in outpatient services utilization and underrepresentation of women in the kidney replacement therapy (KRT) population.

## Methods

### Estimation of the sex distribution among individuals with CKD in the Austrian general population

To estimate the sex distribution among individuals with CKD in Austria, we used electronic medical records (EMRs) of 698,065 patients collected in 58 general practitioners’ offices, spanning from 1989 to 2008, which have been described in detail elsewhere [39]. We included only subjects above 18 years of age, and whose records include at least one creatinine measurement. We then calculated eGFR based on the 2009 CKD-EPI formula, suppressing the race factor (race was not documented), as well as using the race-free 2021 CKD-EPI formula [31]. We classified all subjects into Kidney Disease: Improving Global Outcomes (KDIGO) CKD stage thresholds (no CKD and G1–G2: eGFR ≥ 60, G3: 30 ≤ eGFR < 60, G4: 15 ≤ eGFR < 30, G5: eGFR < 15 ml/min/1.73 m^2^) [40]. Since the data did not include albumin measurements, we were not able to make a distinction between subjects without CKD and the early CKD stages G1 and G2. In the following, we will denote subjects classified to CKD G3–G5 as CKD patients.

### Estimation of the sex distribution among CKD patients treated at outpatient nephrology clinics in Austria

To estimate the sex distribution among known CKD patients, we obtained data from outpatient clinics of six nephrology departments in Austria (Medical University of Vienna, Universitätsklinikum St. Pölten, Keplerklinikum Linz, Klinikum Wels-Grieskirchen, Landeskrankenhaus Villach, Medical University of Innsbruck). Specifically, we collected age in years, sex, and appointment dates. The included outpatients had to be over 18 years of age. Kidney transplant (KTX) and peritoneal dialysis (PD) patients were excluded from the analysis, except for Wels, where from 1409 patients, 159 were on PD or KTX, but as this information was only accessible on aggregate, the respective records could not be identified and thus not excluded. The respective Institutional Review Boards approved this data extraction (EK 1363/2016 Vienna, ECS 1020/2019 St. Pölten, ECS 1128/2019 Linz, Wels, ECS 1066/2020 Innsbruck, MZ 24/19 Villach), and the study adhered to the Declaration of Helsinki.

### Definitions of sex and gender

Following the WHO definition, sex is understood as “different biological and physiological characteristics of females, males and intersex persons”. Gender “refers to the characteristics of women, men, girls and boys that are socially constructed” [41]. Our data included binary sex only. To remain consistent with previous work, subjects with male or female sex are referred to as men and women, respectively.

### Statistical analysis

#### Sex distribution of CKD prevalence among patients from general practitioners’ offices

Age, serum creatinine levels (µmol/l), and eGFR (ml/min/1.73 m^2^) according to the 2009 CKD-EPI and 2021 CKD-EPI formulas were summarized descriptively for the whole dataset and per sex. We used means (SD) or medians [interquartile range] for metric variables, and counts (%) for categorical variables. The number of observations per patient varied widely, further we suspected that patients with problematic kidney function were more likely to be monitored more frequently. To limit this potential bias in repeated measurements, we calculated the mean of the first three (or less) records per patient of all values (age, creatinine, eGFR).

#### CKD outpatient clinic data

Age, number of visits per year, and percentage of men and women among outpatients were summarized descriptively, both for the whole dataset and for individual centers. We used means (SD) or medians [interquartile range] for continuous variables, and counts (percentages) for categorical variables.

**Fig. 1:**
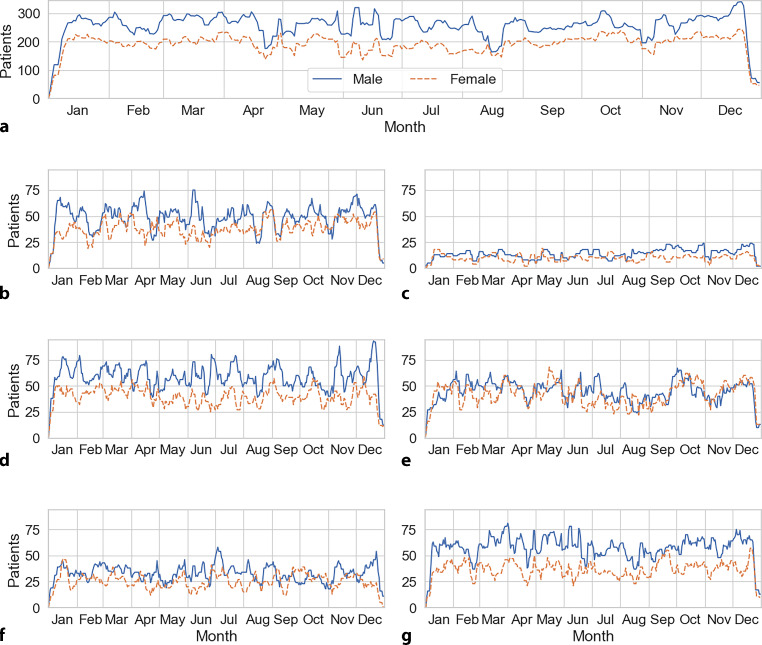
**a**–**g** 7-day moving averages of daily number of men and women outpatients. **a** Shows the averages for all centers, while **b**–**f** show the values for every center. **a** Overall, **b** Innsbruck, **c** Linz, **d** St. Pölten, **e** Vienna, **f** Villach, **g** Wels

The 7‑day moving averages of numbers of male and female outpatients were charted over the course of the year 2019 for both sexes in every center (Fig. 1). To improve readability of the graph, we used 7‑day moving averages to prevent the number of patients falling to zero on weekends (when outpatient clinics are closed).

## Results

### Austrian CKD prevalence: basic patient characteristics and their sex distribution

After exclusion of minors and records without serum creatinine assessment, the final EMR dataset consisted of 129,258 records (42% men, 58% women) from 39,800 patients (42.4% men, 57.6% women). The median age of men was 50.6 [38.9, 64.5] years, while the median age of women was 52.4 [37.5, 68.2] years. Mean serum creatinine was 1.0 ± 0.3 mg/dl for men and 0.9 ± 0.3 mg/dl for women (Table 1).

**Table 1:** Estimated chronic kidney disease (CKD) prevalence in Austria between 1989 and 2008 by sex; eGFR was calculated using the CKD-EPI 2009 [30] and revised 2021 [31] equation. Continuous variables are summarized by mean (SD) or median [IQR] and tested via student’s t or Mann-Whitney‑U tests, categorical variables are summarized by counts (%) and tested via χ^2^-test

	Men 42.4% (*N* = 16,891) | Women 57.6% (*N* = 22,909) Total *N* = 39,800				
		Men	Women	Total	*p* value
*–*	*Age*	50.6 [38.9, 64.5]	52.4 [37.5, 68.2]	51.6 [37.7, 66.6]	< 0.001
*–*	*Creatinine* [mg/dl]	1.0 (0.3)	0.9 (0.3)	0.9 (0.3)	< 0.001
CKD-EPI 2009	*eGFR* [ml/min/1.73 m^2^]	86.9 (19.8)	80.3 (21.2)	83.1 (20.9)	< 0.001
	*CKD stage*	–	–	–	< 0.001
	No CKD and G1–2	15,446 (91.4%)	19,160 (83.6%)	34,606 (86.9%)	–
	G3	1336 (7.9%)	3479 (15.2%)	4815 (12.1%)	–
	G4	87 (0.5%)	226 (1.0%)	313 (0.8%)	–
	G5	22 (0.1%)	44 (0.2%)	66 (0.2%)	–
CKD-EPI 2021	*eGFR* [ml/min/1.73 m^2^]	91.1 (19.4)	84.0 (20.9)	86.0 (20.6)	< 0.001
	*CKD stage*	–	–	–	< 0.001
	No CKD and G1–2	15,865 (93.9%)	20,091 (87.7%)	35,956 (90.3%)	–
	G3	941 (5.6%)	2621 (11.4%)	3562 (8.9%)	–
	G4	68 (0.4%)	157 (0.7%)	255 (0.6%)	–
	G5	17 (0.1%)	40 (0.2%)	55 (0.1%)	–
–	Difference of eGFR_2021_ and eGFR_2009_	4.39 (1.33)	3.9 (1.14)	4.11 (1.25)	< 0.001

*eGFR* estimated glomerular filtration rate, *CKD* chronic kidney disease, *CKD-EPI* CKD-Epidemiology Collaboration

The overall prevalence of CKD in stages G3–G5 was 13.5% using the 2009 CKD-EPI equation (suppressing the race factor [30]) and 10.1% when GFR was estimated using the 2021 CKD-EPI equation (creatinine fit without race [31]). The CKD prevalence was higher for women using both equations and in G3–G5 CKD stages. The overall sex-specific prevalence of CKD in stages G3–G5 was 16.4% for women versus 8.5% for men using the 2009 CKD-EPI equation, and 12.3% for women versus 6.1% for men with the 2021 CKD-EPI equation. Using the 2021 CKD-EPI equation, the eGFR in men was on average 4.39 ml/min/1.73 m^2^ higher than with the 2009 CKD-EPI equation. In women this difference amounted to 3.90 ml/min/1.73 m^2^ (*p* < 0.0001 for the difference between the sexes).

### CKD outpatient clinic visits at six Austrian centers: visit frequency, basic patient characteristics and their sex distribution

Among all 7255 outpatients in 2019 who attended the 6 nephrology outpatient clinics studied in Austria in the year 2019, 45% were women. Women were generally younger (average age 58.9 ± 8.3 years in women, 60.4 ± 17.4 years in men). The median number of visits was two per year for both sexes for patients with at least two visits in 2019 (Table 2).

**Table 2:** Age, number of visits per year and average days between visits from the included outpatients. Shows values for both sexes and overall

		Overall	Women	Men
*n *(%)	**–**	7255	3268 (45.0)	3987 (55.0)
Age in years	Median [Q1,Q3]	62.0 [48.0, 74.0]	61.0 [45.0, 74.0]	63.0 [49.0, 74.0]
Visits per year	Median [Q1,Q3]	2.0 [1.0, 4.0]	2.0 [1.0, 4.0]	2.0 [1.0, 4.0]
Average days between visits (*n* = 4596)^a^	Mean (SD)	67.2 (58.5)	66.7 (58.8)	67.6 (58.3)

^a^Only patients with two or more visits throughout 2019 were included

The per site analysis showed that patients in Vienna were considerably younger (median of 55 years) than in other centers (medians of age between 61 and 67 years). The number of patient visits per year was higher for both St. Pölten and Villach (median of 3 vs. 2 at other centers and 1 in Innsbruck). Among patients with at least two visits, the mean average days between visits showed outliers for Wels (80.9 ± 56.7 days) and Linz (90.9 ± 59.1 days) compared with the overall mean of 67.2 ± 58.5 days (note that as we observed only one year, the average days between visits do not sum up to 1 year.) Overall and center-specific sex distribution of outpatient CKD-patients is shown in Table 3.

**Table 3:** Age, number of visits per year and average days between visits from the included outpatients. Shows sex-aggregated values for each center and percentages for men and women outpatients

		Overall		Linz	St. Pölten	Vienna	Villach	Wels
*n*	**–**	7255	1867	424	1139	1693	726	1409
Age in years	Median [Q1,Q3]	62.0 [47.0, 74.0]	63.0 [50.0, 76.0]	65.0 [53.0, 76.0]	67.0 [53.0, 77.0]	55.0 [39.0, 69.0]	67.0 [52.2, 76.0]	61.0 [47.0, 72.0]
Visits per year	Median [Q1,Q3]	2.0 [1.0, 4.0]	1.0 [1.0, 3.0]	2.0 [2.0, 4.0]	3.0 [2.0, 6.0]	2.0 [1.0, 3.0]	3.0 [2.0, 5.0]	2.0 [1.0, 5.0]
Average days between visits (*n* = 4596)^a^	Mean (SD)	67.2 (58.5)	76.5 (62.9)	90.9 (59.1)	53.4 (48.2)	59.1 (59.6)	53.0 (54.6)	80.9 (56.7)
Sex, *n* (%)	Women	3268 (45.0)	880 (47.1)	170 (40.1)	493 (43.3)	826 (48.8)	334 (46.0)	566 (40.2)
	Men	3987 (55.0)	987 (52.9)	254 (59.9)	646 (56.7)	867 (51.2)	392 (54.0)	843 (59.8)

^a^Only patients with two or more visits throughout 2019 were included

The daily numbers of men compared to women outpatients were higher at every center and overall. Outpatient numbers were lower in the last week of December at all sites (Fig. 1).

## Discussion

To estimate the sex distribution of CKD in Austrian primary care, we analyzed electronic medical records from general physicians during the years 1989–2008. The overall CKD G3–G5 prevalence was 13.5%, using the 2009 CKD-EPI without a correction for race. The CKD G3–G5 prevalence was higher in women (16.8%), compared to men (9.1%). We further analyzed the sex distribution among outpatients of six Austrian nephrology departments. In contrast to the higher CKD prevalence in women, only 45% of nephrology outpatients were women.

The CKD prevalence was higher for women than men for stages G3–5. The eGFR formulas correct for sex to factor in differences between men and women, e.g. higher muscle mass in men, which causes creatinine to be higher and thus eGFR to be lower. The eGFR formulas were evaluated for both sexes and did not show sex-specific differences in estimation bias [30]. The overall CKD prevalence in our study was markedly higher than the estimation of the Austrian CKD prevalence of 9.77% reported in the 2017 Global Burden of Disease Study [33]. This discrepancy is most likely due to the selection bias inherent in our cohort, which only includes records from general practitioners where creatinine was measured, which is more likely to occur e.g. when clinicians suspect kidney disease.

Considering an ongoing discussion about race-free GFR estimation alternatives, we compared the currently most common eGFR formula, 2009 CKD-EPI, to its recently introduced race-free version, 2021 CKD-EPI. Since the new eGFR is higher for individuals of non-black race, the 2021 CKD-EPI equation delivered lower CKD prevalence estimates, most pronounced in stages G3 and G4. The mean difference between the 2009 and 2021 eGFR values was greater for men. The shift in prevalence values from the 2009 to the 2021 formula was in fact greater for women. The new formula was fitted and evaluated in US-based populations and its validity remains to be determined in non-US populations.

In 2019 fewer female outpatients were treated at the six participating nephrology departments in Austria. They were younger than their male counterparts, whereas the number of visits per year was comparable for men and women. Whether these differences reflect the choice of the patients or the modality of the treatment centers of clinicians is unclear, but it is likely that nephrology clinic follow-up appointments are determined by the clinicians rather than the patients. The findings of our study are at odds with publications which show greater health care utilization among women [34–36, 42], albeit not for CKD patients. Interestingly, in the dataset used for the prevalence estimation, which consists of general practitioner medical records, we also observed the same number of general practitioner (GP) visits per sex (median of two visits, throughout the period from 1989 to 2008). In previous cohort analyses, men predominated over women in CKD cohorts from France [43], the USA [44], Japan [45], Japan [46], Germany [47] and China [48]. This tendency was also observed in the CKD outcomes and practice patterns study (CKDopps), where the clinic visit frequency was slightly higher for women than men (2.9 versus 2.6 visits per year for women versus men, respectively [25, 49]).

One could question if the differences in eGFR between men and women are meaningful, because they do not necessarily translate into differences in CKD outcomes. Yet it is well observed that women with CKD have higher excess mortality compared to women in the general population, than men with versus without CKD [50]. Also, large observational studies showed that mortality hazards by eGFR are similar between men and women [51, 52]. Further, in the USA women have been shown to be less aware of impaired kidney function (defined as low eGFR) [53]. In Austria, more men than women initiate dialysis [54], and at least in the past men on dialysis had better access to the waiting list for a deceased donor organ than women [55].

One popular hypothesis trying to explain the sex disparity in CKD treatment is based on biological causes, as men have been shown to have a faster CKD progression than women [56]. It is still unclear if this faster deterioration in men is rooted in biological differences between the sexes, or is caused or amplified by differing lifestyle factors or socioeconomic influences; however, the difference in outpatient visits could be attributed to the condition of male patients being more severe and deteriorating rapidly but given the higher prevalence in women in all CKD stages G3–G5 in Austrian primary care, it is still unexpected to observe more men than women in Austrian nephrology departments. This could indicate that also other factors besides biological differences interfere with proper CKD diagnosis and treatment.

As a strength of the present analysis, the EMR dataset allowed us to assess CKD prevalence in Austrian primary care, based on a large sample size. The prevalence calculation provided additional insights about how the GFR equations influence prevalence estimates. The outpatient analysis is multicenter and country-specific. The latter prevents the results from being confounded with cultural differences or different health care policies.

The prevalence calculation is, however, limited by the fact that this dataset is already 13 years old. Furthermore, the dataset contains selection bias. Healthy persons could have been underrepresented in EMRs from general practitioners’ offices, whereas persons with normal kidney function are underrepresented among patients who had their kidney function parameters measured. Both instances of underrepresentation could have led to overestimation of CKD prevalence.

Our outpatient data are confounded by 156 out of 1409 patients from Wels being either PD or kidney transplant patients. The outpatient clinic in Linz was open on 2 days per week. The number of patients on a given day is in fact estimated by a 7-day moving average, and that is the reason why the numbers are consistently lower in Linz.

One of the questions meriting further investigation is whether the men-to-women ratio among outpatient visits may have shifted over time (e.g. during the last decade). If a shift indeed occurred to the women’s benefit, the hypothesis about sex bias disadvantaging women would be bolstered. Moreover, men and women outpatient visits could be compared with the number of men and women treated as inpatients or outpatients in the last decade. The result could e.g. speak for higher threshold for women to utilize outpatient services, or for fewer women ever receiving specialized inpatient or outpatient nephrology treatment.

Another relevant question is whether the use of the race-free CKD-EPI 2021 equation should be considered for the Austrian population, instead of its previous 2009 iteration. The new formula could overestimate eGFR even more than in the USA, where 13.4% of the population self-identified as black or African American in 2019 [57]. Even though it is questionable how generalizable the findings of the recent evaluations of the new formulas are to non-US populations [58], as stated in the introduction a large part of the Austrian population is very possibly non-black. We fully acknowledge the problem inherent to racial corrections in medical assessments and agree that racial corrections should be avoided wherever possible; however, the low awareness of CKD in the general populations [53], and the difficulty of diagnosing CKD as early as possible to choose optimal treatment, renders the use of a new formula that potentially overestimates eGFR (and thus underestimates CKD prevalence) for the large part of the population, counterproductive. Future research should evaluate the new eGFR formulas in non-US based populations and especially investigate the usability of race-free cystatin-based alternatives.

In summary, this study showed for Austria, in accordance with international data [2–24], that CKD was more prevalent among women than men. More men than women, however, were under nephrological care in all Austrian outpatient clinics analyzed. These results draw attention to the sex disparity that exists in the care for individuals with kidney disease, according to clinicians who participated in a recent interview study [59]. Whether this disparity was also perceived by the patients themselves will be simultaneously reported. Irrespective of patient-perceived impressions, the discrepancy between higher CKD prevalence for women but lower CKD care in dedicated clinics should be aggressively addressed, as it is well known that early care can slow the progression of kidney disease [60, 61].
